# The Effects of Storage Conditions on Lycopene Content and Color of Tomato Hot Pot Sauce

**DOI:** 10.1155/2018/1273907

**Published:** 2018-01-31

**Authors:** He Li, Jian Zhang, Yong Wang, Jian Li, Yihe Yang, Xinqi Liu

**Affiliations:** ^1^Beijing Advanced Innovation Center for Food Nutrition and Human Health, Beijing Technology & Business University, Beijing 100048, China; ^2^Nutrition & Health Research Institute, COFCO Corporation, Beijing Key Laboratory of Nutrition & Health and Food Safety, Beijing 102209, China

## Abstract

Tomato hot pot sauce (THPS) at different storage temperatures (0, 25, and 37°C) and with two kinds of packaging for 120 days was investigated in this study. High performance liquid chromatography was employed for detecting lycopene and 5-hydroxymethylfurfural (HMF). The changes of lycopene and HMF during storage were regressed with kinetic equation of both zero-order and first-order models, and the latter fitted better. The kinetic equation constant (*k* value) of lycopene or HMF at 37°C was higher than that at 25°C. The *k* value of lycopene of PET/PE (P1) packaged THPS was 1.60 times of that of PET/Al/EAA/PE (P2) packaged at 37°C, while it was 2.12 times at 25°C. The *k* value of HMF of P1 packaged THPS was 1.69 times of that of P2 packaged at 37°C, while it was 1.01 times at 25°C. Significant correlations between color index of *L*^⁎^, *a*^⁎^, and *a*^⁎^/*b*^⁎^ and lycopene or HMF were found at storage temperature. Browning color was attributed to both Maillard reaction and degradation of lycopene. In conclusion, lower storage temperature and stronger oxygen barrier property of package could maintain color stability and extend shelf life.

## 1. Introduction

Hot pot is one of the most popular Chinese foods in China and has spread all over the world since it is very easy to keep its authentic taste. The core of hot pot taste is the hot pot sauce, which is used to prepare the base soup of hot pot. In the last decade, hot pot sauce has been upgraded from handmade in small kitchen to industrialized central kitchens which are equipped with large sauce cooking bowl up to 1000 kg/batch. This upgrade not only benefits the hot pot restaurants to uniform the recipe and increase the kitchen efficiency, but also provides consumers with alternative choice to cook top-taste hot pot in their own home. Tomato hot pot sauce (THPS) is made from tomato paste, soybean oil, tomato, onion, ginger, and other seasonings and could be classified as vegetable-based semisolid seasoning. Because of its unique soup color, sour taste, and nutritional value, THPS is growing popular among consumers. The sales of THPS also increase rapidly in supermarkets and wholesale market place; thus it becomes a favored product category by food companies and chain restaurant with central kitchen. But the long time storage on shelf (usually 6–12 months) could lead to unfavorable quality changes such as nutrients degradation or browning color, which might be taken as unsuitable for consumption [1].

There are many studies on the color changes of tomato paste [1], tomato juice [2], tomato powder [3], and tomato sauce [4] during processing and storage. But the color change of THPS during processing and storage remains unclear, and the mechanism behind the color change needs further investigation. Maillard reaction and the ascorbic acid oxidation might be two reasons that contribute to the color change of tomato product during the long-term storage [3]. Lycopene is not only an important characteristic component, but also the major coloring ingredient of tomato and tomato products. Thus lycopene has been a hot topic among the researches on the color changes of tomato products [5], although the changes of amino acid content or reducing sugar are also related to the quality change in storage.

In this study, the quality of THPS was investigated to explore the mechanism of color change during storage. The contents changes of lycopene and 5-hydroxymethylfurfural (HMF) were tracked under different packaging and storage conditions. It is helpful to guide the optimization of packaging and storage conditions of THPS, so as to improve the shelf life of the products and meet the market demand.

## 2. Materials and Methods

### 2.1. Chemicals

Standard lycopene and 5-hydroxymethylfurfural (HMF) were purchased from Sigma-Aldrich Chemical Co. (United States). Ethanol and acetonitrile of HPLC grade were purchased from Fluka Chemical Co. (Germany). Pyrogallic acid, potassium ferrocyanide, zinc sulphate, and other reagents were of analytical grade.

### 2.2. Tomato Hot Pot Sauce and Preparation

Tomato paste (cold break) was purchased from COFCO Tunhe Co., Ltd. (China). Other food materials were purchased from local market.

**Figure 1:**
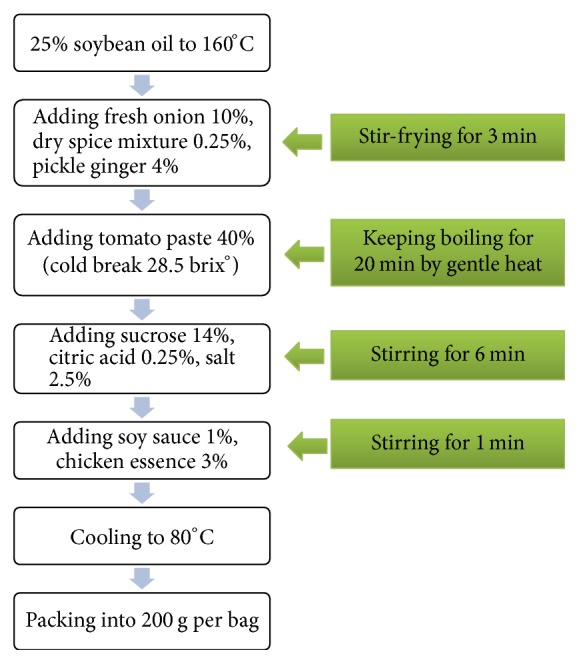
Tomato hot pot sauce making procedures.

The THPS was prepared according to the following procedures. Firstly, the samples were weighed and waited for further cooking, including tomato paste (cold break, 28.5 Brix°) 40%, soybean oil 25%, sucrose 14%, fresh onion 10%, pickle ginger 4%, chicken essence 3%, salt 2.5%, soy sauce 1%, citric acid 0.25%, and dry spice mixture (cinnamon, amomum tsao-ko, clove, aniseed, fennel, white cardamom, bay leaf, dried orange peel, and Chinese red pepper, with equal weight) 0.25%. Secondly, as shown in Figure 1, the soybean oil was heated up to 160°C, followed by adding compound spice, pickle ginger, and onion and stirring for 3 min. Thirdly, the tomato paste was added and the sample was kept at the intermittent boiling state for 20 min by gentle heat. Fourthly, sucrose, citric acid, and salt were added and stirred for 6 min. Fifthly, soy sauce was added together with chicken essence with one-minute stirring. Finally the samples were cooled down to 80°C and packaged into 200 g per bag.

*Storage Conditions*. There were packaging P1 (polyethylene terephthalate/polyethylene), with oxygen permeability 60.00 cm^3^/(m^2^·24 h·0.1 MPa), and packaging P2 (polyethylene terephthalate/aluminum/ethylene acrylic acid copolymer/polyethylene), with oxygen permeability 0.23 cm^3^/(m^2^·24 h·0.1 MPa). The storage temperature was 0°C, 25°C, or 37°C, respectively, and they were stored for 0, 30, 60, 90, or 120 days.

### 2.3. Color Analyses

The color of THPS was measured using a colorimeter of Labscan XE (HunterLab, Hunter Associates Laboratory Inc., United States). A whole package of THPS 200 g was transferred into a beaker. After mixed for 2 minutes at 3000 r/min by a blender, the samples were placed in Petri dishes and filled to the top. Color was recorded as *L*^*∗*^ (lightness), *a*^*∗*^ (green-red tonality), and *b*^*∗*^ (blue-yellow tonality). The Hue value (*a*^*∗*^/*b*^*∗*^) was calculated based on measured, *a*^*∗*^ and *b*^*∗*^, values [1].

### 2.4. Lycopene Analysis

Briefly, a whole package of THPS 200 g was transferred into a beaker. After mixed for 2 minutes at 3000 r/min by a blender, 2.000 g THPS was weighed in a 250 mL flask and wrapped with aluminum foil to prevent exposure to light. The procedure of extraction and HPLC detection was in accordance with previous study [6]. 50 mL of solvent (50 : 25 : 25 hexane/acetone/ethanol containing 2.5% pyrogallic acid) was added with nitrogen protection. After shaken for 10 min, 10 mL of distilled water was added for a further shaking. The hexane phase was filtered through a 0.2 *μ*m nylon filter. One injection volume of filtrate 10 *μ*L was injected into a liquid chromatography equipped with diode array detector (LC-Prominence-20AT and SPD-M20A, Shimadzu Co., Japan). An analytical column C_18_ (Shim–pack VP-ODS 15 cm × 4.6 mm ID, 5 *µ*m) was employed and kept at 30°C. An isocratic elution of mobile phase with 50 : 50 methanol/acetonitrile was delivered at a flow rate 1 mL/min. Lycopene was detected at 472 nm, and its calibration curves (*R*^2^ = 0.984) had previously been established by the standard lycopene. The limit of detection was 2.6 × 10^−6^ *µ*g/mL, the recovery rate was 92%, and coefficient of variation was 3.44%. The peaks and areas were calculated with LC solution software.

### 2.5. 5-Hydroxymethylfurfural Analysis

After homogenization of the sample, 5-hydroxymethylfurfural concentration was determined by HPLC [6, 7]. 5.000 g of THPS was placed in a 50 mL centrifuge tube, and 2 mL of 15% (w/v) potassium ferrocyanide and 2 mL of 30% (w/v) zinc sulphate were added with slow stirring and the volume made up with distilled water. After standing for 30 min, the mixture was centrifuged for 1 h at 12,000 r/min. Then 2 mL supernatant was filtered through a 0.2 *μ*m cellulose acetate filter. HPLC apparatus, column, injection volume of sample, and software of chromatograms analysis were the same as lycopene employed in the above part. The column was kept in a stabilizer at 40°C, and an isocratic elution of mobile phase with 90 : 10 water/methanol was delivered at a flow rate 1 mL/min. HMF was detected at 285 nm, and its calibration curves (*R*^2^ = 0.992) had previously been established by the standard HMF. The limit of detection was 1.9 × 10^−4^ *µ*g/mL, the recovery rate was 91%, and coefficient of variation was 4.22%.

### 2.6. Ascorbic Acid Analysis

Ascorbic acid concentration was determined by HPLC [8].

### 2.7. Statistical Analysis

All experiments were performed in triplicate. Statistical analyses were performed with SPSS 11.5. The results were expressed as the means ± standard deviation (SD) of triplicate. The data were subjected to one-way analysis of variance (ANOVA) and the significance of difference between samples means was calculated by Duncans' multiple range test.* P *< 0.05 indicates the significant difference. Pearson correlation test was used to analyze the correlation between lycopene, HMF, and color index.

The reaction model of the relationship between the content change and the time was analyzed by the zero-order equation [9, 10] (1) or the first-order equation [11–14] (2), and the correlation coefficient *R*^2^.

(1) y=C−kt

(2) y=C∗exp−kt,

where “*y*” is the dependent variable of lycopene, HMF, or color index; “*t*” is the time; “*k*” is the kinetic equation constant; and “*C*” is the starting value.

## 3. Results and Discussion 

### 3.1. Changes of Lycopene Content

**Figure 2:**
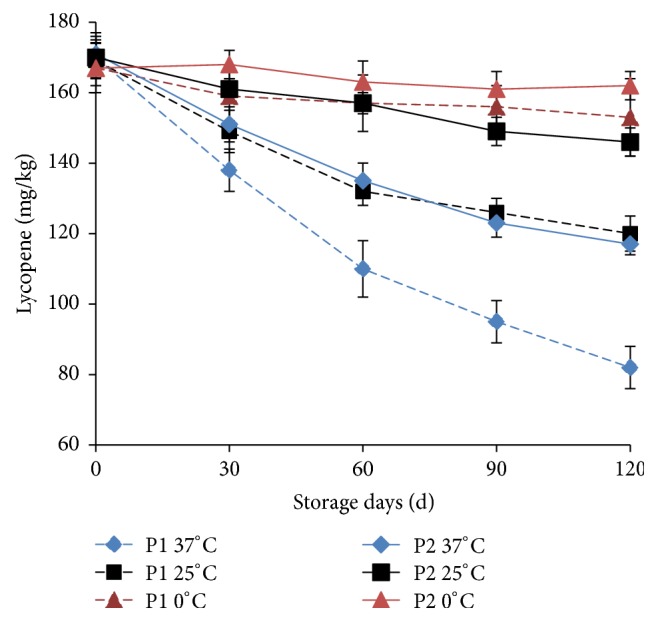
Changes of lycopene in THPS during storage. The experiments were performed in triplicate, and results were expressed as the means ± standard deviation (SD).

Lycopene is an important characteristic nutrient substance in tomato, and it is also the main coloring material of tomato. Thus it is of practical significance to study the changes of lycopene content during storage period. As shown in Figure 2, the content of lycopene did not change significantly during the storage of the two types of packaging (P1 and P2) under the storage conditions of 0°C (*P* > 0.05). At 25°C and 37°C, the contents of lycopene in the two types of packaging (P1 and P2) decreased with the prolongation of storage. After storage for 30 days, the content of lycopene at 37°C was significantly lower than that of the same packaging at 25°C (*P* < 0.05). Tamburini et al. [15] found that there was no change in lycopene content of tomato purée during one year's storage. A similar result was found, and no change was observed in lycopene content of tomato ketchup during 8 months of storage at 30°C [6]. The stability of lycopene might be attributed to the thermal inactivation of enzymes that might expose lycopene to oxidants by destroying the cell wall.

In this study, the moisture content of THPS was low and the oil content was close to 20%. During the high temperature frying process, the tomato lycopene was dissolved from the cell wall and transferred to the oil and exposed to the oxidizing environment. During the storage period it could further extend the lycopene degradation [16–18]. Oxygen permeability rate of P2 packaging was much lower than that of P1, thus reducing the lycopene oxidation loss. As shown in Figure 2, the content of lycopene in the P2 packaged THPS was significantly higher than that of the same storage time in P1 packaged sample (*P* < 0.05) at 25°C or 37°C for 30 days.

The fitting results of the zero-order kinetic model and the first-order kinetic model were shown in Table 1. The lycopene loss rate of the two types of packaged THPS under the conditions of 25 and 37°C storage was well fitted with the zero-order kinetic and the first-order kinetic model equation, where *R*^2^ of the first-order equation was higher than that of the zero-order equation. The relationship between lycopene and the time-dependent change in THPS was more consistent with the first-order kinetic model equation. The degradation of free lycopene and encapsulated lycopene in the storage model system also showed that lycopene degradation was accorded with the first-order kinetic model, and the kinetic constants increased with raising storage temperature [12]. It has also been reported that the degradation of free lycopene content was at a lower rate in oil media comparing with water media [13].

As shown in Table 1,* k* value of first-order kinetic model at 37°C was 1.86 times of the *k* value at 25°C for P1 packaged THPS, while it was 2.46 times for P2 packaged THPS. Because of the lower oxygen permeability, packaging P2 showed a greater effect on suppressing degradation of lycopene than packaging P1. When THPS was stored at 37°C or 25°C, the *k* value of P1 packaged THPS was 1.60 or 2.12 times of the *k* value of P2 packaged THPS, respectively. It could be seen that the storage temperature and the oxygen permeability of the packaging had great influence on the degradation of lycopene in THPS.

### 3.2. Changes of HMF Content

**Figure 3:**
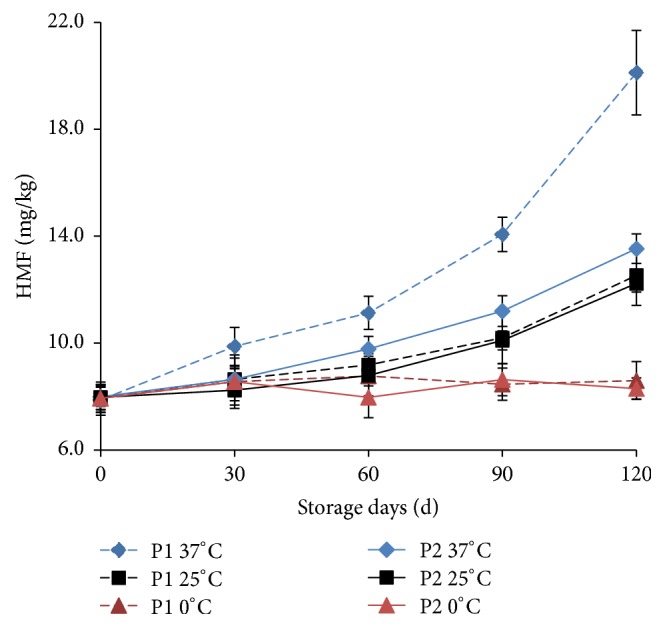
Changes of HMF in THPS during storage. The experiments were performed in triplicate, and results were expressed as the means ± standard deviation (SD).

HMF is a product of the Maillard reaction at early stage, and the HMF content can be used as a measure of the extent of the Maillard reaction [19]. HMF is promoted to produce brown nitrogen-containing polymers, making the product color deterioration for a longer storage time. As shown in Figure 3, the content of HMF in the THPS of P1 and P2 package was not significantly changed (*P* > 0.05) at 0°C within the storage period. At 25°C and 37°C, the contents of HMF in the THPS of P1 and P2 package increased with the storage time.

Storage temperature was an important factor in affecting the reaction speed of Maillard reaction. The HMF content of THPS with P1 package was significantly higher if stored at 37°C than that stored at 25°C, when both samples were stored for more than 30 days. The same rule of HMF content of THPS with P2 package was found, when samples were stored for more than 60 days. Packaging could affect the amount of HMF in THPS during whole storage period. But in this study, it was found that the HMF content in THPS was significantly different (*P* < 0.05) between P1 package and P2 package, when THPS was stored at a high temperature (37°C) and more than 60 days.

The fitting results of HMF content and storage time by using zero-order kinetic model and first-order kinetic model were shown in Table 2. The results showed that the growth of HMF content was in accordance with the zero-order kinetics and the first-order kinetic model equation. And the coefficient of determination (*R*^2^) of first-order kinetic equation was higher than that of the zero-order kinetic model at 25°C and 37°C storage. Thus the first-order kinetic model equation was used to analyze the relationship between the content of HMF in THPS and storage time. Similarly, in a kheer mix powder storage model, the formation of HMF followed a first-order reaction at 37 or 45°C also and showed a good correlation [14].

As shown in Table 2, the kinetic equation constant (*k* value) of HMF at 37°C was higher than that at 25°C. The *k* value of HMF of P1 packaged THPS was 1.69 times of that by P2 packaged at 37°C, while it was 1.01 times at 25°C. It could be seen that there was no significant difference in the content of HMF in P1 and P2 package at 25°C (*P* > 0.05). But when the storage temperature was high (37°C), the P2 package was better than P1 package to inhibit the increase of HMF content.

### 3.3. Changes of Ascorbic Acid Contents

Ascorbic acid oxidation can also cause nonenzymatic browning. During storage, the ascorbic acid oxidation degradation is often dependent on the processing method and the storage conditions. Ascorbic acid degradation followed first-order kinetic equation in strawberry jam during storage, and the rate constant (*k*) increased with an increase in the temperature [20]. As the TPHS is usually subjected to a long period of boiling, ascorbic acid oxidation degradation is promoted and will cause the sample color to become brown. The content of ascorbic acid before storage was less than 1.0 mg/kg, and there was no significant change at 0, 25, and 37°C during storage (*P* > 0.05). It showed that the ascorbic acid was almost all destroyed during the thermal processing.

### 3.4. Changes of Color Index

**Figure 4:**
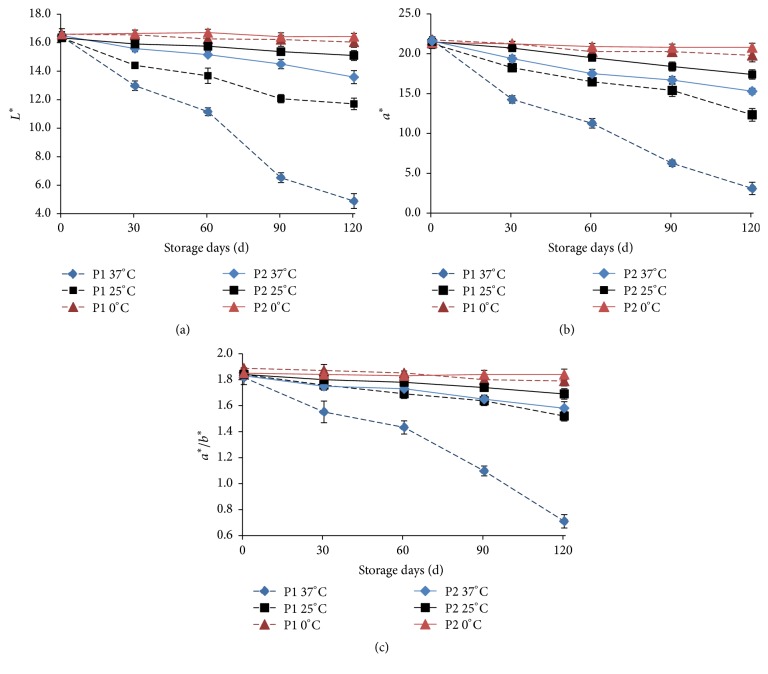
Changes of *L*^*∗*^ (a), *a*^*∗*^ (b), *a*^*∗*^/*b*^*∗*^ (c) in THPS during storage. The experiments were performed in triplicate, and results were expressed as the means ± standard deviation (SD).

The change in color can be used to evaluate the shelf life of the THPS, which is one of the most important indicators of the THPS quality or other tomato products [21, 22]. In order to highlight the change of the redness of THPS, the color constant *a*^*∗*^/*b*^*∗*^ value is introduced to better evaluate the color [1]. Both *a*^*∗*^/*b*^*∗*^ value and *a*^*∗*^ value are used as an important index of international trade in tomato products. The higher the *L*^*∗*^, *a*^*∗*^, and *a*^*∗*^/*b*^*∗*^ value of the tomato products, the more acceptable the color [3, 6]. In Figures 4(a), 4(b), and 4(c), it could be seen that there was no significant difference in *L*^*∗*^, *a*^*∗*^, *a*^*∗*^/*b*^*∗*^ between P1 and P2 at 0°C (*P* > 0.05). At 25°C or 37°C, the values of *L*^*∗*^, *a*^*∗*^, or *a*^*∗*^/*b*^*∗*^ in the TPHS were decreased with the storage time prolonged. This was consistent with the decrease of lycopene content and the increase of HMF content.

When the THPS in P1 package was stored at 37°C after 30 days, the *L*^*∗*^ value was significantly lower than that at 25°C (*P* < 0.05). But a significantly different *L*^*∗*^ value of P2 package at 37°C and 25°C was found after 60 days of storage (*P* < 0.05). It was also found that the *L*^*∗*^ value of the P2 packaged TPHS at 37°C was still higher than the *L*^*∗*^ value of the same storage period at 25°C with P1 package (Figure 4(a)). When the THPS in P1 package or P2 package was stored at 37°C for more than 30 days, the *a*^*∗*^ value was significantly lower than that at 25°C (*P* <0.05). The values of *a*^*∗*^ of P2 package THPS storage at 37°C were significantly higher than those of the P1 packaged THPS at 25°C (Figure 4(b)). The value of *a*^*∗*^/*b*^*∗*^ of P1 packaged THPS storage at 37°C after 30 days was significantly lower (*P* < 0.05) than that at 25°C. But a significantly different *a*^*∗*^/*b*^*∗*^ value of P2 package at 37°C and 25°C was found after 60 days of storage (*P* < 0.05). There was no significant difference in the *a*^*∗*^/*b*^*∗*^ value between THPS stored at 37°C in P2 package and at 25°C in P1 package for 60 or 90 days (*P *> 0.05). When the THPS was stored for 120 days, it was found that the *a*^*∗*^/*b*^*∗*^ value of the P2 packaged TPHS stored at 37°C was significantly higher (*P* < 0.05) than that of the P1 packaged TPHS stored at 25°C (Figure 4(c)). As a result, the P2 package reduced the deterioration of the color index compared to the P1 package. Even if the storage temperature rose to 37°C, the color index of P2 sample has equal degree of reduction to the P1 sample at 25°C.

HMF content can reflect the browning of processed fruit and vegetable products during storage, thus affecting the *L*^*∗*^, *a*^*∗*^, and *a*^*∗*^/*b*^*∗*^ of the THPS. As shown in Table 3, the Pearson correlation coefficients of HMF and *L*^*∗*^, *a*^*∗*^, *a*^*∗*^/*b*^*∗*^ were significantly negatively correlated (*r* = −0.881~−0.988) under different storage temperatures and packaging types. Thus the loss of *L*^*∗*^, *a*^*∗*^, and *a*^*∗*^/*b*^*∗*^ was mainly caused by Millard reaction, which is consistent with the changes HMF presented above [3]. The degradation of lycopene mainly produces ketones, aldehydes, alcohols, furan, olefins, aromatics, and a small amount of acids and esters [23]. There was a significant positive correlation (indicated by Pearson correlation coefficient) between lycopene and *L*^*∗*^, *a*^*∗*^, *a*^*∗*^/*b*^*∗*^ under different storage temperature and packaging (*r* = 0.929–0.995). Differently, it was not found that the change of lycopene was significant during 8-month storage in tomato ketchup [6] and 5-month storage in tomato powder [3]. The stability of lycopene might be attributed to the thermal inactivation of enzymes that might expose lycopene to oxidants by destroying the cell wall [24]. However, it is known that THPS is processed by high temperature soybean oil, and part of lycopene is extracted from cell wall and dissolved in oil. In this study, lycopene of THPS shows a poor stability unless placed in oxygen resistance packaging and kept in low temperature storage. Therefore, the HMF content which could be used as index of the Maillard reaction and the content of lycopene had significant effect on the color change of THPS during storage. During the storage period, the control of lycopene content decrease and the HMF content increase can effectively maintain the color of THPS.

## 4. Conclusions

When the storage temperature was 25°C and 37°C, the lycopene and color index (*L*^*∗*^, *a*^*∗*^, *a*^*∗*^/*b*^*∗*^) of the two kinds of packaged THPS were significantly decreased (*P* < 0.05), while the HMF content was increased (*P* < 0.05). The changes of the above parameters were not significant at 0°C (*P *> 0.05). The changes of lycopene and HMF during the storage of THPS can be fitted with the first-order equation. At 25°C and 37°C, the degradation of color index (*L*^*∗*^, *a*^*∗*^, *a*^*∗*^/*b*^*∗*^), the decrease of lycopene content, and the increase of HMF content all showed similar trends, indicated by Pearson correlation coefficient.

Low temperature and high oxygen resistance packaging can reduce the increase in HMF and lycopene reduction, slow down the storage process of browning, protect lycopene, and improve the color of THPS at the end of the storage period. The *L*^*∗*^, *a*^*∗*^, *a*^*∗*^/*b*^*∗*^ of oxygen resistance packaging is also approximately the same as or better than that of the composite film at 25°C. The effect of oxygen resistance packaging on extending shelf life can be more obvious than that of composite film if THPS is stored under unfavorable high temperature. 30 kinds of polyphenols in tomato [25], which may transfer into tomato paste and THPS, will play a role in inhibition of lycopene oxidation. The effects of antioxidant polyphenols on lycopene protection should be investigated in further studies. These pieces of information could be used to guide the processing and storage of THPS.

## Figures and Tables

**Table 1:** Kinetic equation of lycopene in THPS during storage.

Storage temperature	Packaging	Kinetic equation		Correlation coefficient *R*^2^
37°C	P1	First-order	*y*_1_ = 166exp(−0.00512*t*)	0.956
		Zero-order	*y*_1_ = − 0.729*t* + 163	0.936
	P2	First-order	*y*_1_ = 167exp(−0.00320*t*)	0.942
		Zero-order	*y*_1_ = − 0.453*t* + 167	0.926

25°C	P1	First-order	*y*_1_ = 164exp(−0.00276*t*)	0.900
		Zero-order	*y*_1_ = − 0.463*t* + 164	0.885
	P2	First-order	*y*_1_ = 169exp(−0.00130*t*)	0.862
		Zero-order	*y*_1_ = − 0.199*t* + 169	0.859

*Note*. “*y*_1_” is the dependent variable of lycopene (mg/kg); “*t*” is storage time (day).

**Table 2:** Kinetic equation of HMF in THPS during storage.

Storage temperature	Packaging	Kinetic equation		Correlation coefficient *R*^2^
37°C	P1	First-order	*y*_2_ = 7.66exp(0.00741*t*)	0.962
		Zero-order	*y*_2_ = 0.0957*t* + 6.88	0.908
	P2	First-order	*y*_2_ = 7.72exp(0.00438*t*)	0.939
		Zero-order	*y*_2_ = 0.0455*t* + 7.50	0.914

25°C	P1	First-order	*y*_2_ = 7.74exp(0.00359*t*)	0.938
		Zero-order	*y*_2_ = 0.0359*t* + 7.57	0.902
	P2	First-order	*y*_2_ = 7.55exp(0.00354*t*)	0.867
		Zero-order	*y*_2_ = 0.0346*t* + 7.38	0.846

*Note*. “*y*_2_” is the dependent variable of HMF (mg/kg); “*t*” is storage time (day).

**Table 3:** Correlation analysis of *L*^*∗*^, *a*^*∗*^, *a*^*∗*^/*b*^*∗*^ with lycopene and HMF in THPS during storage.

Color index	Packaging	Storage temperature	Pearson correlation coefficient (*r*)	
			Lycopene	HMF
*L* ^*∗*^	P1	37°C	0.963^*∗∗*^	−0.934^*∗*^
		25°C	0.976^*∗∗*^	−0.881^*∗*^
	P2	37°C	0.967^*∗∗*^	−0.967^*∗∗*^
		25°C	0.995^*∗∗*^	−0.926^*∗*^

*a* ^*∗*^	P1	37°C	0.992^*∗∗*^	−0.916^*∗*^
		25°C	0.962^*∗∗*^	−0.951^*∗*^
	P2	37°C	0.994^*∗∗*^	−0.931^*∗*^
		25°C	0.981^*∗∗*^	−0.942^*∗*^

*a* ^*∗*^/*b*^*∗*^	P1	37°C	0.929^*∗*^	−0.988^*∗∗*^
		25°C	0.943^*∗*^	−0.974^*∗∗*^
	P2	37°C	0.958^*∗*^	−0.975^*∗∗*^
		25°C	0.974^*∗∗*^	−0.966^*∗∗*^

*Note*. *∗∗* indicates an extremely significant correlation with *P *< 0.01; *∗* indicates a significant correlation with *P *< 0.05.
